# The relevance of contact-independent cell-to-cell transfer of TDP-43 and SOD1 in amyotrophic lateral sclerosis

**DOI:** 10.1016/j.bbadis.2017.07.007

**Published:** 2017-11

**Authors:** Maya A. Hanspal, Christopher M. Dobson, Justin J. Yerbury, Janet R. Kumita

**Affiliations:** a Department of Chemistry, University of Cambridge, Lensfield Road, Cambridge CB2 1EW, UK; b Illawarra Health and Medical Research Institute (IHMRI), University of Wollongong, NSW 2522, Australia

**Keywords:** ALS, amyotrophic lateral sclerosis, CFTR, cystic fibrosis transmembrane conductance regulator, CNS, central nervous system, CSF, cerebrospinal fluid, fALS, familial ALS, FTLD, frontotemporal lobar degeneration, LMN, lower motor neurons, sALS, sporadic ALS, UMN, upper motor neurons, WT, wild-type, MND, motor neurone disease, SOD1, copper-zinc superoxide dismutase 1, FUS, fused in sarcoma/translocated in liposarcoma, TDP-43, Tar DNA-binding protein 43, ThT, Thioflavin T, Conditioned medium, TDP-43, SOD1, Spreading, Aggregates

## Abstract

Amyotrophic lateral sclerosis (ALS) is a neurodegenerative disease involving the formation of cytoplasmic aggregates by proteins including TDP-43 and SOD1, in affected cells in the central nervous system (CNS). Pathology spreads from an initial site of onset to contiguous anatomical regions. There is evidence that for disease-associated proteins, including TDP-43 and SOD1, non-native protein conformers can promote misfolding of the natively folded counterparts, and cell-to-cell transfer of pathological aggregates may underlie the spread of the disease throughout the CNS. A variety of studies have demonstrated that SOD1 is released by neuron-like cells into the surrounding culture medium, either in their free state or encapsulated in extracellular vesicles such as exosomes. Extracellular SOD1 can then be internalised by naïve cells incubated in this conditioned medium, leading to the misfolding and aggregation of endogenous intracellular SOD1; an effect that propagates over serial passages. A similar phenomenon has also been observed with other proteins associated with protein misfolding and progressive neurological disorders, including tau, α-synuclein and both mammalian and yeast prions. Conditioned media experiments using TDP-43 have been less conclusive, with evidence for this protein undergoing intercellular transfer being less straightforward. In this review, we describe the properties of TDP-43 and SOD1 and look at the evidence for their respective abilities to participate in cell-to-cell transfer via conditioned medium, and discuss how variations in the nature of cell-to-cell transfer suggests that a number of different mechanisms are involved in the spreading of pathology in ALS.

## Highlights

• Protein aggregates transfer between cells in motor neuron disease.
• Cell contact-independent mechanisms may be a route of transfer.
• SOD1 undergoes cell-to-cell transfer via conditioned medium in cell culture.
• It is still unclear whether TDP-43 consistently undergoes cell-to-cell transfer
• Differences between the two proteins may explain this observation.

## 1 Amyotrophic lateral sclerosis and protein misfolding

Amyotrophic lateral sclerosis (ALS), the most common form of motor neurone disease (MND), is a rapidly progressive neurodegenerative condition caused by the loss of upper (UMN) and lower motor neurons (LMN) in the central nervous system (CNS). This neuronal loss results in a range of symptoms that may include muscle weakness and wasting, limb rigidity and difficulty in swallowing, speaking and breathing, culminating in paralysis and eventually death. Symptoms usually present at a focal site of onset, often an upper or lower limb, before spreading to contiguous neuroanatomical regions [1], [2]. The extension of clinical symptoms is accompanied by the spatiotemporal progression of pathology throughout the CNS [3], [4], [5], [6], which is characterised by the deposition of aggregated proteins in affected cells. Familial ALS (fALS) accounts for approximately 10% of all cases of the disease, with the other 90% being sporadic (sALS); however, there is no evident difference in clinical presentation between sALS and fALS patients, suggesting that a common molecular mechanism underlies the disease. Misfolded Tar DNA-binding protein 43 (TDP-43) is the major component of the inclusions found by autopsy in the majority of both sALS and fALS patients, although in a small fraction of cases the inclusions primarily contain the proteins copper-zinc superoxide dismutase 1 (SOD1) or fused in sarcoma/translocated in liposarcoma (FUS) [7].

### 1.1 TDP-43

TDP-43 is a protein containing 414 amino acid residues and is encoded by the TARDBP gene. It belongs to a family of RNA binding proteins known as heterogeneous nuclear ribonucleoproteins (hnRNPs), but, unlike most members of this family, TDP-43 forms a homodimer even in the absence of RNA [8], [9]. It has a range of physiological roles involving the control of transcription and translation, including splicing and the regulation of non-coding RNA [10]. TDP-43 regulates its own mRNA levels through binding to the 3’UTR of the TDP-43 transcript, such that high levels of protein downregulate endogenous TDP-43 expression via a negative feedback loop. Only one ALS-associated mutation, occurring in a region potentially important for RNA binding, has been found to interfere with this feedback loop [11]. It is possible, however, that TDP-43 aggregates could also disrupt this process by sequestering functional protein, thus removing regulatory control over TDP-43 production [11].

The accumulation of aggregated and post-translationally modified TDP-43 is considered a biochemical signature of ALS, and may play a significant role in driving pathogenesis. It was first identified as a component of inclusions in both sALS and frontotemporal lobar dementia (FTLD) by two different groups in 2006, having previously been known to bind a motif in the HIV transactive response DNA, and to participate in a complex involved in the splicing of the cystic fibrosis transmembrane conductance regulator (CFTR) gene [12], [13], [14]. Since then, much work has been done to investigate the way in which TDP-43 contributes to the onset and progression of ALS. Over 50 disease-associated mutations have been identified [10], [15], the majority being located within an aggregation-prone region of the protein and appearing to enhance its aggregation propensity [16]. Under normal conditions TDP-43 is predominantly a nuclear protein, but in ALS and FTLD there is a loss of functional TDP-43 which results in detrimental effects on RNA metabolism, in combination with the cytosolic accumulation of aggregates that leads to neurotoxicity [17], [18]. TDP-43 aggregation culminates in the deposition of both round and skein-like cytoplasmic inclusions, often associated with depletion of the protein from the nucleus [13], [14].

TDP-43 contains two RNA recognition motifs (RRM1 and RRM2), which interact with nucleic acids, as well as a glycine-rich C-terminus. RRM1 is responsible for preferential binding of TDP-43 to UG-repeated motifs in RNA [19]. Almost all ALS-associated mutations in TDP-43 occur in the C-terminus within the glycine-rich domain, strongly suggesting that the function of this domain is important in neurodegeneration [20]. The C-terminus not only mediates protein-protein interactions but also contains a region rich in glutamine (Q) and arginine (N) residues (Q/N rich region), which confers properties similar to those of yeast prions [21]. The similarity in sequence between the Q/N-rich region and yeast prion domains may relate to its cellular function, as when the prion-like domain within the C-terminus of full-length TDP-43 tagged with GFP was replaced with the yeast prion domain of Sup35 in transfected cells, *in vivo* splicing assays showed that this construct regulated exon 9 skipping of the (CFTR) gene in the same way as WT TDP-43 does [16]. This observation suggests that the Q/N-rich region may have a functional role under normal conditions and only adopts an aggregation-prone conformation after a disease-triggering event [21]. The general consensus, based on a number of studies designed to pinpoint the key sequences for aggregation, suggest the critical sequence for aggregation is located between residues 286–353 [22], although there is evidence that the truncated C-terminal RRM2 domain (208–265) drives formation of non-amyloid fibrils, as confirmed by the amyloid detecting dye Thioflavin T (ThT), *in vitro* and *in vivo* [9]. While aggregates formed from C-terminal fragments or the full-length protein do not react with Congo Red, another dye used to confirm that aggregates are amyloid [23], two recent studies have found that the fluorescent dye Thioflavin-S, that also binds to amyloid species, binds to TDP-43 positive skein-like inclusions in a subset of ALS cases, suggesting that pathological TDP-43 aggregates may in fact have amyloidogenic properties [24], [25]. Studies using ALS patient brain lysate containing pathogenic TDP-43 have shown that this material can seed the formation of insoluble TDP-43 inclusions in cell cultures, and this can propagate between cells over serial passages [26], [27].

### 1.2 SOD1

The gene that encodes copper-zinc superoxide dismutase 1 (SOD1) was first associated with ALS in 1993, with mutations in this gene accounting for 20% of fALS cases [28], [29]. Its canonical role is as a free radical scavenger, but there is emerging evidence that it may also act as a transcription factor [30] and an RNA binding protein [31]. It is highly stable in its native state, but may misfold in response to a variety of triggers, including oxidative stress. Nearly 200 disease-causative mutations have been identified throughout the sequence, all of which appear to converge on the exposure of hydrophobic surfaces that are normally buried within the molecule when natively folded [32]. Misfolding of SOD1 results in a gain of function effect, with the presence of aggregates leading to cell death, and SOD1-ALS pathology is characterised by the deposition of conglomerate inclusions enriched in SOD1 that are normally negatively immunoreactive for TDP-43 [33].

Eukaryotic WT SOD1 exists as a stable 32 kDa homodimer predominantly located in the cytoplasm, although it can also be found in the nucleus [34]. The homodimer adopts an eight-stranded Greek key β-barrel structure stabilised by an intra-subunit disulphide bond, and each subunit binds one copper atom and one zinc ion. These post-translational modifications all contribute to the stability of SOD1, making it exceptionally resistant to heat denaturation, and conferring the ability to retain function under denaturing conditions [35], [36].

It is thought that immature and unfolded SOD1, in a disulphide-reduced state that does not bind zinc or copper ions, is responsible for aggregation *in vivo*, although identifying the structure of the species involved is challenging due to the conformational heterogeneity of its non-native state. ALS-associated mutations, as well as aberrant post-translational modifications to the WT protein, destabilise SOD1 and cause it to misfold [37]. It is currently unclear whether or not amyloid fibrils are important in SOD1 mediated ALS, as SOD1 positive inclusions from ALS patient tissue do not show Thioflavin-S or Congo Red binding [23]. SOD1 does, however, bind to ThT during *in vitro* aggregation assays [38]. Furthermore, computational modelling has identified amyloidogenic segments within the molecule that can be used to produce synthetic peptides that aggregate into structures with fibrillar morphology when imaged using transmission electron microscopy [39].

*In vitro* aggregation studies have been carried out with WT SOD1 and several mutational variants, under a range of different conditions to characterise the different types of aggregate species. Initial experiments used the apo WT SOD1 variant where the cysteine residues at positions 6 and 11 were substituted by alanine and serine respectively (apo AS-WT SOD1) and showed this protein has similarities to WT SOD1 in terms of structure, stability and metal binding. It was found to be possible to form fibrils from soluble apo AS-WT SOD1 at pH 3.5 in the presence of EDTA over a period of months and electron microscopy showed aggregates of mixed morphology including fibrils [40]. These conditions, however, are not close to physiological, and so further work has been done to explore aggregation under conditions more likely to be encountered inside a cell. Apo SOD1 containing an N-terminal His_6_ tag and where cysteine 6 and 11 were both mutated to serine (SS-SOD1) was observed to form insoluble amyloid fibrils at pH 7 and apo SOD1 where all four cysteines are reduced, thereby lacking the disulphide bond, was observed to form aggregates in physiological buffer conditions at 37 °C with agitation overnight. These aggregates exhibit amyloid properties when analysed using a range of biophysical techniques and the fibrils were also found to seed the aggregation of soluble SOD1 [41]. Apo WT human SOD1 recombinantly expressed in yeast, thereby retaining all post-translational modifications occurring *in vivo*, has also been studied and observed to form fibrillar species under mild reducing conditions. It was also found that the presence of a small amount of disulphide reduced apo WT SOD1 was sufficient to induce fibril formation of apo WT SOD1, suggesting that destabilisation of the homodimer produces monomeric SOD1 nuclei that recruits apo WT SOD1 into the fibrils, providing a role for WT SOD1 in the disease process [42].

Different SOD1 mutational variants have previously been observed to have a variety of aggregation propensities when expressed in cultured cells, enabling the behaviour of recombinant SOD1 variants *in vitro* to be compared to their intracellular aggregation [37]. Using motor-neuron like NSC-34 cells expressing different SOD1 variants tagged with eGFP, it was possible to quantify the number of aggregates present 48 h post-transfection. By plotting the end point ThT fluorescence versus the percentage of cells showing inclusions for each variant, it was evident that the aggregation propensity of SOD1 *in vitro* can predict its aggregation propensity in cells. Interestingly, the strongest correlation was between the *in vitro* aggregation propensity for specific variants and patient disease duration, which could be used to predict disease severity. It appears that the aggregation kinetics governing *in vitro* fibril formation and aggregation in mouse models are very similar, although this does not directly translate to human disease [43]. Nevertheless, these strategies have provided a great deal of information regarding the folding and aggregation of SOD1 in disease.

SOD1 also participates in the cell-to-cell propagation of aggregates over serial passages [44], [45], suggesting that protein aggregation represents an important and common pathogenic mechanism. Many mutations in genes encoding proteins other than TDP-43 and SOD1 have since been discovered, but histopathology mainly converges on the deposition of misfolded TDP-43, and in some cases, SOD1 or FUS, regardless of the initial cause of the disease [29].

### 1.3 Other molecular players in ALS and FTLD: FUS, C9orf72 and tau

FUS is another RNA binding protein that is normally found in the nucleus but shows enhanced aggregation propensity as a result of ALS-associated mutations, and is deposited in cytoplasmic inclusions in a small subset of both sALS and fALS cases (for review see [46]). One study has demonstrated that a mutational variant of FUS can seed the aggregation of WT FUS both *in vitro* and *in vivo*; however, there is currently no experimental evidence of cell-to-cell transfer of FUS aggregates in a similar manner to those of TDP-43 and SOD1 [47].

A major discovery was made in 2011, when genomic sequencing revealed a noncoding GGGGCC nucleotide repeat expansion in the first intron of gene C9orf72 as the most common cause of familial ALS and FTLD, as well as accounting for a considerable proportion of sporadic cases [48], [49]. While the presence of fewer than 30 repeats is now associated with disease, in affected patients this can reach up to hundreds or even thousands of repeats, although there is no clear relationship between the size of the nucleotide repeat expansion and either disease severity or age of onset [50]. Putative mechanisms of neurotoxicity include: loss of functional C9orf72 protein, production of toxic RNA foci and accumulation of different dipeptide repeats encoded by the hexanucleotide repeat expansions (for review see [50]), all of which are capable of disrupting normal cellular function. Very recently it was shown that these dipeptide repeat proteins can undergo cell-to-cell transfer through direct intercellular contact as well as exosome dependent mechanisms [51].

No mutations in the MAPT gene encoding the microtubule-associated protein tau have so far been associated with ALS, but there appears to be an overlap in histopathology between hyperphosphorylated tau and TDP-43 in some cases of ALS with cognitive impairment, although the significance of this is unclear [52]. Tau pathology is more commonly found in diseases including Alzheimer's disease and a subset of FTLD, collectively termed as tauopathies. Transfer of aggregated tau between cells has been demonstrated in several studies (for review see [53]), and there is evidence that different tau strains exist that are capable of serially propagating their distinct structural properties in both cell lines and in mice.

### 1.4 Protein misfolding and aggregation in disease

Protein misfolding and aggregation has been implicated in a number of increasingly prevalent human diseases ranging from neurodegeneration to diabetes [54]. The ability of a normally soluble peptide or protein to aggregate into ordered fibrils has been discovered to be a generic property of polypeptide chains, although aggregation propensity is, in part, encoded in the amino acid sequence [55]. Persistent protein misfolding *in vivo* arises either from natively folded proteins adopting an alternative conformation or through incorrect folding during synthesis [56]. This is a comparatively rare event as there are cellular strategies in place either to refold the protein into a functional structure or to degrade misfolded conformers. Partially folded or misfolded proteins, however, pose a problem because they are at greater risk of aberrant interactions with other molecules. This is because hydrophobic regions of the protein that are normally buried within the core of the molecule become exposed to the aqueous environment and can self-associate through hydrophobic interactions and hydrogen bonding, thereby losing solubility associated with the native state [54]. Protein aggregates can adopt a variety of stable structures including amorphous assemblies, oligomeric species and amyloid fibrils. The latter species are of particular interest because they are highly structured self-associated protein multimers with characteristic physico-chemical properties.

Despite the number of proteins linked with misfolding diseases, there are no common features of their primary sequences or their secondary or tertiary structures [54]. Amyloid fibrils themselves share a remarkably similar structural nature, forming closely packed structures rich in β-sheet content. They form long thread-like filamentous structures that are approximately 10 nm in diameter, but can reach micrometers in length, with mature fibrils consisting of intertwining protofibrils. Their core architecture involves a cross-β structure that is highly stable due to the arrangement of backbone hydrogen bonding, which is a property shared by all polypeptide sequences. Amyloid fibrils may in fact be more thermodynamically stable than natively folded states, even under physiological conditions. As the conversion from soluble protein to fibrils requires intermolecular contacts, the thermodynamic stability of fibrils is increased at higher protein concentrations [55].

**Fig. 1:**
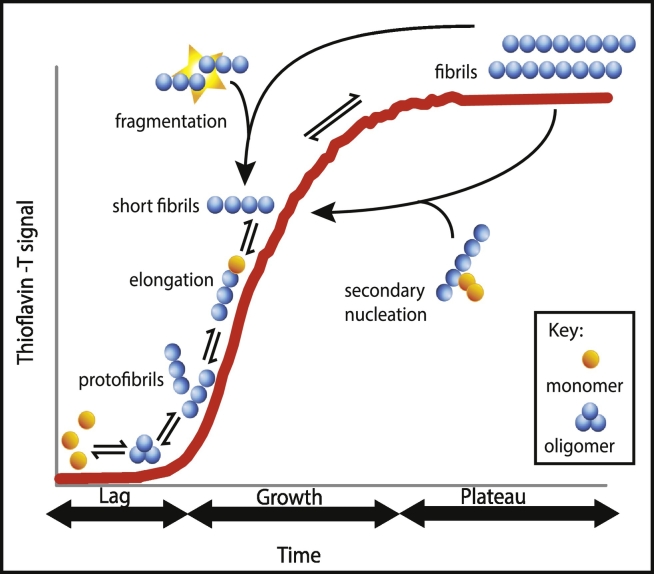
Schematic representation of the kinetics of amyloid formation. Amyloid formation is characterised by an initial lag phase in which monomeric species nucleate and form oligomeric species followed by a growth phase of fibril formation, which occurs through elongation and secondary events (fragmentation and/or secondary nucleation). A plateau is reached when amyloid formation is in a steady state as free monomers (i.e. protein molecules) are no longer available.

Analysing the kinetics of amyloid formation has yielded a great deal of information on the process of converting soluble natively folded proteins to amyloid fibrils (Fig. 1). In the presence of a limited quantity of monomeric soluble protein, the reaction typically follows a sigmoidal curve over time, defined by a slow lag phase followed by a rapid growth phase, and then reaching a plateau once the pool of monomers is depleted. Soluble monomeric proteins initially polymerise through nucleation mechanisms generating oligomers, species that then form protofibrils, and finally mature fibrils. The rapid growth phase involves elongation of the aggregate by the addition of free monomers to the fibril ends, and also secondary processes, such as the fragmentation of fibrils to increase the number of growing ends, or via secondary nucleation in which a fibril acts as a catalytic site for free monomers to aggregate. These mechanisms contribute to the generation of more aggregates, demonstrating that amyloid growth is capable of rapid proliferation following the initial nucleation process [55].

Like in other neurodegenerative disorders, including Alzheimer's and Parkinson's disease, the presence of aggregates is linked to ALS pathology; each condition is characterised by the progressive and gradual spread of an aggregated signature protein deposited in inclusions in a stereotypical pattern through the neuroaxis [57]. These observed generic characteristics have led to the emergent concept of ‘prion-like behaviour’ as a mechanism underlying the aggregation and spread of misfolded proteins in these diseases, which is a conceptual framework borrowed from prion diseases [56], [57], [58]. The mammalian cellular prion protein, PrP^c^, is a glycosyl-phosphatidylinositol anchored membrane-bound glycoprotein encoded by the PRNP gene that can misfold into the β-sheet rich pathological conformer PrP^Sc^. What makes the prion unique is not its capacity to self-propagate through induced misfolding and amplification in an amyloidogenic manner, reflected by an incubation period of several years followed by a rapid clinical presentation phase, but by its transmissibility between individuals and, exceptionally, across species [59]. Such infectivity appears to occur without RNA or DNA driving replication, unlike conventional infectious agents such as bacteria or viruses, with the term ‘protein only hypothesis’ sometimes used to refer to the causative agent of prion diseases [60]. To propagate pathology throughout the CNS, the pathological conformer is thought to leave an affected cell and to be taken up by surrounding cells, where it induces the conversion (also known as ‘seeding’) of endogenous natively folded protein counterparts into the pathological conformation, ultimately causing misfolding and aggregation to spread [61].

Both mammalian and yeast prions share the ability to form amyloid fibrils, but while the former appears to be pathogenic, the latter are normally functional and can even be advantageous in yeast. Out of 20,000 human protein-encoding genes analysed, 240 genes have been identified as containing sequences related to yeast prion-like domains, which are typically enriched in polar, uncharged amino acids [62]. Thirty percent of these are RNA binding proteins, including TDP-43 and FUS, indicating that these low complexity sequences are important for function under normal physiological conditions [62]. The prion-like domain, in particular of TDP-43, confers a high aggregation propensity [16], which may explain why many proteins containing these sequences may misfold and aggregate in neurodegenerative diseases. SOD1 does not contain a prion-like domain, but shares many attributes with mammalian prions, including self-replication *in vitro* and transfer of aggregates in culture [32].

Prion-like propagation is rapidly gaining popularity as an explanation for the progressive intercellular spread of misfolded protein, but it does not entirely explain how proteins that are expressed throughout the nervous system during our life span can start to misfold and aggregate in specific regions, before pathology disseminates to more distant areas [63]. The ‘selective vulnerability’ hypothesis puts forward the idea that certain neuronal populations within the CNS are more susceptible to insults than others, and as these stresses accumulate over time, they induce the misfolding and aggregation of endogenous protein in these cells. Stress signals are transmitted to other populations through diffusible extracellular factors or directly through trans-synaptic signaling, which causes aggregation in less susceptible populations [64]. It is very likely that these mechanisms operate in combination in neurodegenerative disease, making it imperative that we work to fully characterise the process of cell-to-cell aggregate transfer and its relevance to neurodegenerative disease.

**Fig. 2:**
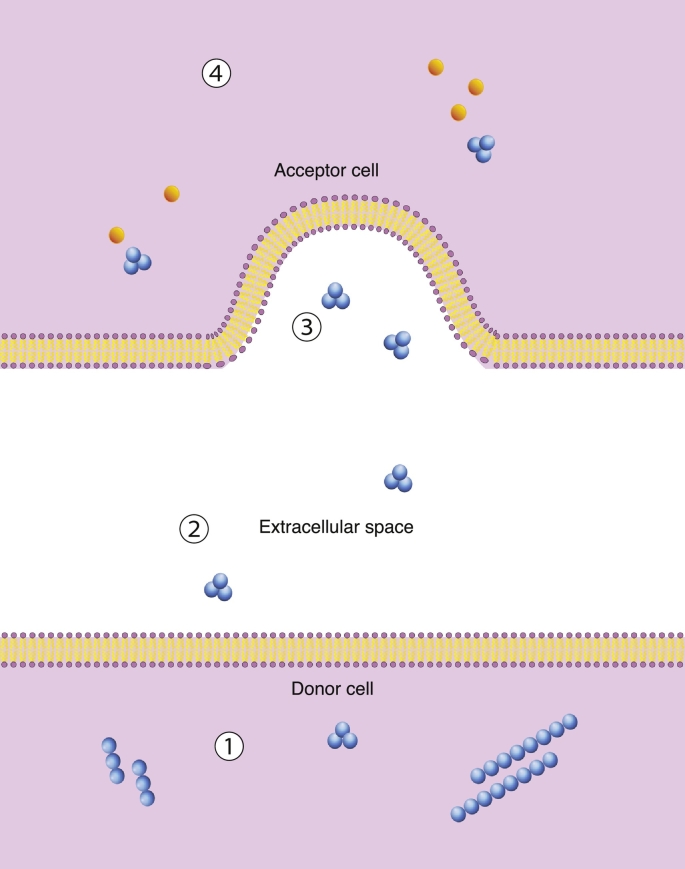
Diagram showing cell-to-cell transfer. The pathological propagative species must pass through the plasma membrane of the donor cell (1) to enter the extracellular environment (2), pass through the plasma membrane of the recipient cell (3) and then induce the conversion of non-pathological protein into its pathological counterpart (4). In this diagram, the propagative species is represented by an oligomeric species; however, the exact structural nature of the transmissible species is not conclusively known.

Several subcellular mechanisms may contribute to cell-to-cell transmission of aggregates, but the process broadly depends on four events: 1) an aggregate passing across the membrane of the donor cell, 2) traversing the extracellular environment, 3) crossing the membrane of the recipient cell and 4) interacting with endogenous natively folded conformers and inducing misfolding [61], [64]. The mammalian prion overcomes the first two stages as it is exposed to the extracellular environment from its location on the plasma membrane, and is secreted under normal physiological conditions. Most pathogenic misfolding proteins, however, are normally cytosolic, or in the case of TDP-43, shuttle between the nucleus and cytoplasm [64]. Several non-classical routes of entry have been suggested, some of which depend on direct contact between cells (tunneling nanotubes, transynaptic contacts), and some of which are contact independent (exosomes, naked aggregates) (Fig. 2) [65], [66], [67] (reviewed in [61]).

Cell-to-cell transfer of aggregates is less well-characterised in ALS compared to other neurodegenerative disorders such as Alzheimer's or Parkinson's diseases, but there remains a strong possibility that prion-like mechanisms are relevant to the pathogenesis of the disease (reviewed in [68]). Sporadic ALS patients have significantly elevated levels of either TDP-43 or SOD1 in their cerebrospinal fluid (CSF) compared to age matched controls, especially at earlier stages of the disease, which indicates that the proteins are released and circulate around the body during the disease process [69], [70], [71]. Exosomes, small endocytic vesicles that are secreted by most cell types into circulating fluids including the CSF, are already implicated with the spreading of a variety of pathological misfolded proteins [72]. Even cultured cells release exosomes and other secreted factors directly into the culture medium, allowing material to accumulate in the extracellular environment over time [73]. Collecting medium from cells overexpressing a misfolding protein, here referred to as conditioned medium, and applying it to a fresh cell culture in order to observe internalisation, provides an important strategy for modelling contact-independent intercellular spread *in vitro*. Such experiments have been conducted in neuron-like cell cultures using both TDP-43 and SOD1. SOD1 appears to undergo cell-to-cell transfer via conditioned medium, and even propagates over serial passages in culture [66]. In contrast, however, those investigating this route for TDP-43 have not been able to demonstrate conclusively that it undergoes cell-to-cell transfer in the same way [27], [74], [75].

In the next section of this review, we describe the evidence of cell-to-cell transfer via conditioned medium for a number of neurodegenerative diseases. We then describe the properties of SOD1 and TDP-43 and compare their respective abilities to participate in cell-to-cell transfer via conditioned medium based on the evidence so far, with a focus on experiments investigating neuron-to-neuron transfer in immortalised cell lines. We go on to discuss how these differences in routes of cell-to-cell transfer may implicate different mechanisms involved in the spreading of pathology in ALS.

## 2 Conditioned medium as a route for cell-to-cell transfer

The experimental paradigm of conditioned medium is dependent on a population of cells in culture that secrete factors into the extracellular environment (known as a donor population). A population of cells without prior exposure to the secreted material (the acceptor population) is then cultured in the conditioned medium to observe whether or not the material originating from the donor population can be internalised by the acceptor cells. Using conditioned medium provides a simpler system to explore cell-to-cell transfer than *in vivo* models, as well as providing much greater control over the experimental conditions.

**Fig. 3:**
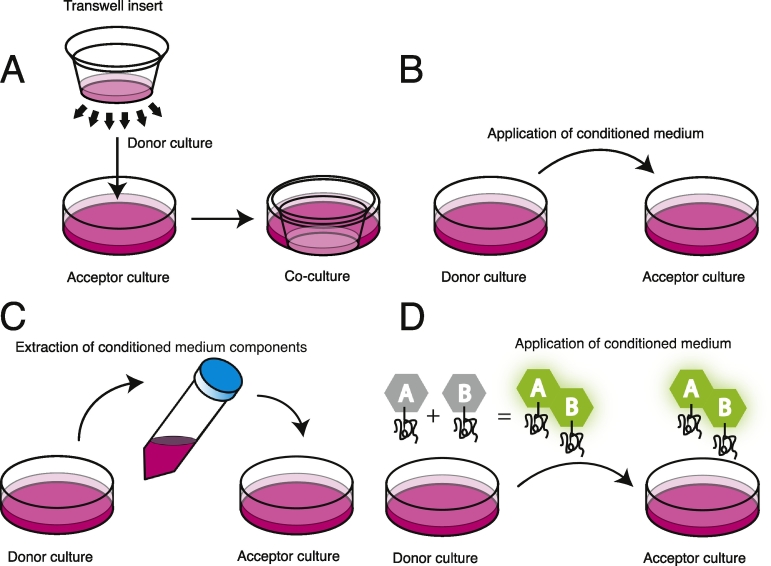
Different conditioned medium experimental procedures. 1) A porous membrane barrier prevents direct contact between the donor and acceptor cultures, allowing only secreted factors of a particular size in the medium to pass between the two compartments 2) Medium from the donor culture is collected and a naïve acceptor culture is incubated in the conditioned medium 3) Before applying the medium from the donor culture to the acceptor culture, components of the conditioned medium are extracted or concentrated 4) The donor culture co-express protein labelled with one of two complimentary fragments that luminesce when in close proximity with one another, which is dependent on self-association of at least two proteins labelled with one of each fragment. Application of conditioned medium obtained from the donor culture onto the acceptor culture will result in bioluminescence in acceptor cells only if the luminescent aggregate species are taken up by the acceptor culture.

Conditioned medium experiments can be categorised according to one of four strategies (Fig. 3):

1) The donor and acceptor cultures are separated by a porous membrane or barrier to prevent cell-to-cell contact,
2) The conditioned medium is collected from donor cells and applied to the acceptor culture,
3) Components, such as exosomes, of the conditioned medium are isolated and applied to the acceptor culture,
4) Complementary protein assay models, which employ a bioluminescent complementary fragment reporter system, such as luciferase, to label the protein of interest in order to report on its aggregation state in conditioned medium. The protein of interest is labelled with one of two complementary fragments. Bioluminescence only occurs if the two complementary fragments are in close enough proximity to fold into the full functional label, which is dependent on at least two complementary labelled proteins associating with one another, indicating the formation of dimers, oligomers or larger aggregates. The conditioned medium is collected from donor cells co-expressing the protein with each label and is applied to the acceptor culture.

## 3 Evidence of cell-to-cell transfer of misfolding proteins through conditioned medium experiments

Cell models have used a variety of different mammalian prion strains, in combination with immortalised and primary neuronal and non-neuronal cell lines, to explore the mechanisms of prion release and uptake between cells [59]. A strain is defined by a specific conformation that is adopted by the normal cellular PrP^C^ prion during the conversion process to the aberrant scrapie PrP^Sc^ prion, which is then faithfully and stably propagated in a host [60]. This, in part, determines the pathological and biophysical properties of a particular strain. Several studies have demonstrated that mammalian prion strains can propagate between neighbouring cells using conditioned medium, some in association with exosomes, but this may depend on the properties of the strain used as well as the cell type. For example, the use of neuron-like Neuro-2A cells overexpressing the mouse adapted scrapie prion strain 22 L shows cell-to-cell transfer via conditioned medium, but the same cell line overexpressing another mouse adapted scrapie prion strain (RML) shows no or little transfer through the same route [76], [77], [78] (for a full review of cell models of mammalian prion strain propagation, see [79]). Building on experiments carried out on mammalian prion strains in conditioned medium, researchers have been able to demonstrate cell-to-cell transfer of many other aggregation-prone proteins involved in neurodegenerative diseases using similar experimental procedures, a variety of aggregate species and different immortalised neuron-like cell lines (Table 1).

**Table 1:** Examples of misfolding proteins involved in neurodegenerative diseases that undergo cell-to-cell transfer in different conditioned medium experimental set ups. N.A., not applicable, N.C., not characterised.

Experimental set up	Protein of interest	Disease	Transferring species	Cell line
Membrane barrier	Dipeptide repeat proteins from C9orf72 hexanucleotide repeat expansions	ALS	N.C.	NSC-34 [51]
	SOD1	ALS	N.C.	Neuro 2 A [45]
Application onto acceptor culture	Tau	Alzheimer's	N.C [80]., Fibrils [81]	HEK293 [80], Primary neurons [81]
	Huntingtin (Q19 and Q103)	Huntington's	N.C.	HEK donors and SH-SY5Y acceptors [82]
	α-Synuclein	Parkinson's	Monomers, oligomers and fibrils [83] High and low molecular weight oligomers [84]	SH-SY5Y donors and COS-7 acceptors [83] Differentiated and undifferentiated SH-SY5Y [84]
	SOD1	ALS	N.C [45].	Neuro 2 A [45]
	TDP-43	ALS	N.C.	HEK293 [27], [74]
Exosome isolation	Yeast prion Sup35 (NM domain)	N.A.	Multimers [85] N.C [86].	Neuro2A [85], [86]
	SOD1	ALS	N.C.	NSC-34 [66]
Complementary protein assay model	Tau	Alzheimer's	N.C.	HEK293T [87]
	TDP-43	ALS	Dimers, oligomers	HEK293 [75]

## 4 Conditioned medium experiments using SOD1 or TDP-43

### 4.1 Conditioned medium experiments using SOD1

Earlier experiments using pre-formed fluorescently labelled variant SOD1^H46R^ aggregates revealed that these aggregates could be endocytosed by Neuro 2A cells when exogenously applied to the culture medium [45]. These aggregates were capable of converting intracellular SOD1^H46R^ in transfected Neuro 2A cells into puncta that persisted in culture even after the exogenous aggregates had been degraded. Cell-to-cell transfer of different fluorescently labelled aggregates was shown to occur between populations inoculated separately before mixing, even when separated by a 0.4 μm filter membrane, showing that cell-to-cell contact was not necessary for the transfer of these aggregates [45]. Further experiments involving a full-length variant SOD1 (SOD1^G85R^), a C-terminally truncated SOD1 variant (SOD1^G127X^, containing the dominantly inherited G127insTGGG mutation) and WT SOD1 have demonstrated that conditioned medium can propagate aggregates between cells [66], [88]. In addition, misfolded SOD1^G127X^ can interact with intracellular WT SOD1 in NSC-34 cells, converting it to a misfolded conformation by the formation of non-native SOD1 interchain disulphide bonds [88]. Moreover, overexpression of WT SOD1 in cell culture induces misfolding of a proportion of this intracellular SOD1, suggesting that a spontaneous misfolding event may trigger sALS through induced conversion of natively folded SOD1 by a pathological conformer [88]. The discovery that WT SOD1 is also involved in misfolding and aggregation is particularly significant, as this implicates it as a common pathological mechanism irrespective of whether ALS is sporadic or familial [44].

Several GFP labelled SOD1 variants (SOD^A4V^, SOD^G93A^ and SOD^G127X^), as well as human WT SOD1-GFP, transiently overexpressed in NSC-34 cells caused increases in cell death 72 h post-transfection [66]. Western blot analysis and filter trap assays identified oligomeric aggregates (larger than the 0.2 μm membrane pore size) present in the culture medium, including those of WT SOD1, suggesting that dying cells can release aggregates directly into the extracellular environment. However, this cell line is also known to release exosomes, and in stably transfected cells overexpressing different SOD1 variants, exosomes exposed misfolded SOD1 onto the surface of the membrane, in contrast to natively folded WT SOD1, which is normally localised in the lumen [44], [66], [89].

Conditioned medium was collected from human embryonic kidney cells, HEK293, transiently transfected with SOD1 (SOD^G85R^, SOD^G127X^ or WT SOD1) 48 h post-transfection, as these cells displayed resistance to misfolded SOD1 toxicity [66]. Naïve recipient cells expressing endogenous WT SOD1 were cultured in conditioned medium containing variant or WT SOD1 to determine whether or not seeding could be observed. Seeding was in fact serially propagated between cultures over five passages, providing very strong evidence for the prion-like propagation of misfolded SOD1 as a competent seed [66]. Together, this shows that both pathways dependent and independent on exosomes are involved in cell-to-cell transfer using conditioned medium. Exosome independent SOD1 aggregates are taken up via macropinocytosis, a form of non-selective endocytosis characterised by ruffling in the plasma membrane, resulting in the engulfment of large fluid-phase molecules into vesicles [90]. The exosome independent uptake of SOD1 involves a mechanism specific to SOD1 but is not aggregate specific, as soluble SOD1 species are internalised with the same efficiency, suggesting that this process is receptor mediated [89]. Experiments with a microglial cell line have already shown that scavenger receptors participate in selective uptake of SOD1 [91].

### 4.2 TDP-43 in conditioned medium experiments

The SOD1 serial propagation model was also used to investigate whether or not there is a cross-seeding effect between TDP-43 and SOD1 through conditioned medium [74]. The culture medium of HEK293 cells transiently transfected with TDP-43 was collected after 48 h and centrifuged to remove cell debris, as with previous experiments. WT TDP-43 and a variant lacking the nuclear localisation signal (TDP-43^ΔNLS^), causing it to remain in the cytoplasm and undergo aggregation, were used. This medium was then supplemented with 25% fresh complete medium and applied to naïve cells for a 20 h incubation period. Quantitative immunoprecipitation confirmed that 20–40% of detectable endogenous WT SOD1 was misfolded in the lysate of cells incubated with either WT TDP-43 or TDP-43^ΔNLS^. A 15–25 fold drop in WT SOD1 occurred when endogenous SOD1 was knocked down in these cells, excluding the possibility that misfolded WT SOD1 was transferred from the original donor culture through the conditioned medium.

Using the same model, the question as to whether or not TDP-43 pathology was being propagated to recipient cells alongside the misfolded SOD1 was investigated. The over-expressed TDP-43^ΔNLS^ exhibited many of the pathological markers associated with TDP-43 in ALS, including the clear generation of C-terminal truncated fragments (CTFs) and hyperphosphorylated TDP-43 found exclusively in cytoplasmic puncta. These markers, however, were not found in endogenously expressed TDP-43 in the naïve recipient cells incubated in the conditioned medium during the same 20 h time period, suggesting that the pathological TDP-43 could not confer its properties onto the endogenous intracellular TDP-43 in the same manner.

Another recently published study described the application of exogenous seeding material, either the insoluble fraction of TDP-43 derived from HEK293 cells transfected with WT FLAG-tagged TDP-43 (WT FL TDP-43) or ALS patient tissue, via chemical transfection methods to acceptor HEK293 cells over-expressing WT FL TDP-43 [27]. The recipient cells showed increased levels of phosphorylated TDP-43 in response to exposure to both the cell and patient derived seeds, with the WT FL TDP-43 seeds showing a greater effect than the ALS patient inoculum, as demonstrated by immunocytochemistry and western blotting. This effect was further increased after serial passaging, using a subsequent set of recipient cells re-transfected with inoculum derived from the first set of re-transfected cells. However, when conditioned medium collected from cells re-transfected with the seeds was incubated with naïve cells for 72 h, no cell-to-cell transfer was observed.

To date, there is only one reported cell model that demonstrates TDP-43 transferring between cells via conditioned medium. This particular model uses a protein complementation technique based on a two-part luciferase reporter system to quantify intracellular dimers and oligomers, as determined by size exclusion chromatography [75]. TDP-43 was fused to the N-terminal or C-terminal fragment of a luciferase (TDP-L1 and TDP-L2 respectively) and bioluminescence can only occur if these two constructs are both expressed and the proteins are interacting. Thus, the bioluminescence detected as a consequence of the interaction between the two luciferase fragments can be used to report on the aggregation status of the intracellular TDP-43. HEK293 cells were co-transfected with these constructs and the conditioned medium was collected after 60 h. Bioluminescence was detected in the conditioned medium, indicating that TDP-43 was present. When the conditioned medium was applied to naïve non-transfected HEK293 acceptor cells for 72 h, luciferase activity was detected in the acceptor cells even after repeated washing, showing that they were able to internalise the dimers or oligomers present in the medium. A major advantage of this strategy compared to other seeding experiments involving TDP-43 is that the TDP-43 is spontaneously internalised by recipient cells, rather than relying on lipofection to introduce the seed, which is less physiologically applicable to the disease process.

## 5 Issues to be resolved in conditioned medium experiments using TDP-43

Considering the many similarities between SOD1 and TDP-43, which both show some evidence of prion-like propagation *in vitro*, it is surprising to find that one misfolded protein is so readily transmissible through conditioned medium, while the other appears unable to participate as efficiently. Differences in the properties of the two proteins, as well as in the experimental parameters of the various spreading models, may all contribute to explaining why cell-to-cell TDP-43 transfer has not been conclusively observed so far, with the exception of the complementary protein assay model [75]. SOD1 and TDP-43 inclusions rarely co-localise in sALS, and there is experimental evidence in cell models suggesting that SOD1 and TDP-43 are sequestered into distinct inclusions through different pathways [92], which could mean that these two proteins are involved in separate disease mechanisms. More recent evidence, showing that both WT TDP-43 and TDP-43^ΔNLS^ can induce WT SOD1 aggregation in cell cultures via conditioned medium instead implies that there is a mechanistic overlap between the two proteins, and there may be other reasons why there is no clear evidence for cell-to-cell transfer of TDP-43 using conditioned medium [74].

A variety of explanations for the differences have been proposed, including variations in the incubation periods, that the concentration of secreted TDP-43 is too low for uptake or subsequent seeding inside acceptor cells, the aggregates are not stable, or that TDP-43 transfer may be exclusively dependent on cell-to-cell contact [27]. The SOD1 model for conditioned medium transfer incubated the donor culture in medium for 48 h post-transfection before collecting, and incubated the acceptor culture for a further 20 h [66]. Using the same experimental set up, it was reported that TDP-43 did not undergo cell-to-cell transfer under the same time frame [74]. In the WT FL TDP-43 model it was unclear how long the donor culture was incubated before it was applied to the acceptor cells; therefore, although the incubation period for the acceptor culture was 72 h, the initial incubation period may not have been sufficient for TDP-43 to accumulate in the medium [27]. The only TDP-43 model that reports spreading via conditioned medium incubated the donor culture for 60 h before applying this medium to naïve cells for a further 72 h, so it may be that TDP-43 takes longer to be released and taken up at appreciable concentrations to allow the observation of internalisation of TDP-43 in acceptor cells [75].

Interestingly, in the studies that did not observe the transfer of TDP-43 via conditioned medium, the levels of TDP-43 present within the medium were not quantified, whereas these levels were confirmed in the study involving the protein complementation-based model monitored with bioluminescence [75]. Immunodetection techniques, including immunoprecipitation, could be used to determine whether TDP-43 is present at detectable levels. Even if TDP-43 is secreted by donor cells, it may be that the levels are too low in the reported models to be detected in recipient cells. Concentrating the medium, or isolating exosomes as has been done using SOD1 conditioned medium, may provide better conditions for spreading through this route. TDP-43 uptake has been reported in several studies, but apart from the model using the protein complementation assay, most have relied on chemical transfection methods to facilitate internalisation, which is less physiologically relevant [22], [26], [27], [75]. On the other hand, it is possible that the luciferase tags in the complementation assay cause perturbations to the conformation or stability of putative TDP-43 oligomers, so that the propagative species in this model may not be formed in the other systems.

It is also possible that TDP-43 only undergoes cell-to-cell transfer through close contact between cells, but the fact that incubating WT SOD1 expressing recipient HEK293 cells with conditioned medium containing WT TDP-43, but not WT FUS, can induce SOD1 misfolding suggests that TDP-43 has an effect without intercellular contacts [74]. Furthermore, TDP-43 is found at elevated levels in CSF from ALS patients, providing a route by which TDP-43 pathology can disseminate over long distances within the CNS [70]. Different routes may have different efficiencies, with direct contact facilitating greater TDP-43 transfer than conditioned medium, but they may operate together rather than exclusively. Two recent models were used to observe spontaneous cell-to-cell transfer of TDP-43, one using turboGFP tagged TDP-43 variant G249A in NSC-34 culture, and the other employed SH-SY5Y cells stably expressing HA tagged WT TDP-43 as the donor population [93], [94]. In both cases, transfer took place under co-culture conditions, and crucially neither use transfection to induce the transfer. These models may provide a good foundation for further exploring the contribution of conditioned medium versus cell contact using TDP-43.

A significant hindrance to understanding fully the role of TDP-43 in ALS has been a lack of structural information for the full-length protein due to its high aggregation propensity. Recent studies, however, have provided detailed structural insights into the individual domains of TDP-43 at the atomic level using both X-ray crystallography and solution NMR techniques to demonstrate the folded nature of the N-terminal and RRM domains and an intrinsically disordered C-terminal region [95], [96], [97], [98], [99]. This information has paved the way for insights into the structural dynamics of TDP-43 and also investigations of potential intra- and inter-molecular contacts that are likely to be pivotal for elucidating the structural nature of the aggregate species that are formed as part of the disease process.

Furthermore, it is unclear which aggregated species are disease relevant, as there are conflicting reports regarding the importance of amyloid fibrils in the disease process. Two studies have found Thioflavin S positive TDP-43 inclusions in a small subset of post-mortem patient tissue, and another has shown that a synthetic peptide based on an aggregation-prone region of the C-terminus forms ThT positive fibrils *in vitro* that are capable of seeding aggregation and show neurotoxicity in cell cultures, suggesting that pathological TDP-43 has amyloid-like properties [22], [24], [25], [100]. In contrast, overexpression of full-length and the C-terminal fragment of TDP-43 in purified *E. coli* inclusion bodies results in the formation of amorphous aggregates, which lack any signatures of amyloid, but which are also highly toxic when present in the cytosol of cells [101]. Recently, there has been a shift in favour of the idea that smaller oligomeric species, rather than stable mature amyloid structures, are the main driver of neuropathology in neurodegenerative diseases [102], [103]. This, however, poses additional challenges in determining which species are most relevant to the disease, as oligomers represent a very heterogeneous subset of aggregates in terms of size and structure [104].

Another factor to consider is the possibility that the properties of propagation and toxicity are not linked, meaning that species that are capable of cell-to-cell transfer may differ from the neurotoxic conformers, as is the case with mammalian prions [105], [106]. For example, highly toxic species may reduce infectivity by causing neuronal cell death during the early stages of amyloid formation, terminating the process before the production of more infectious aggregates. Fibril rigidity may also affect these properties, as more stable amyloid aggregates are less likely to undergo fragmentation, generating fewer ‘seeds’ for further induced misfolding of monomers [55]. For two of the studies, the ‘seed’ was derived from clinically confirmed ALS patient tissue showing TDP-43 positive inclusions, but the aggregates present have not been robustly characterised using biophysical techniques, and there is scope for heterogeneity between and within patient samples [27], [107].

The complexities of understanding the underlying mechanisms of the spread of disease in ALS and other neurodegenerative diseases, is of paramount importance to allow the development of effective therapeutic interventions. A number of robust techniques have been developed in recent years to tackle these questions, and it is clear that in systems for which we are able to gain insight into the structural attributes of the putative seeding aggregates, it is possible to begin to elucidate the mechanisms of cell-to-cell transfer. It is therefore clear that it is imperative to identify the disease-relevant aggregate species in the case of TDP-43 associated ALS, in order to understand fully how propagation within the CNS takes place under disease conditions. Given the recent focus of many groups on defining the structural attributes of TDP-43 in both its physiological and pathological states, we believe it is possible to gain a fuller understanding of the relevance of amyloid and other aggregate species to the disease progression in ALS.

## Transparency document

Transparency document. Image 1
